# Locomotive syndrome is associated with body composition and cardiometabolic disorders in elderly Japanese women

**DOI:** 10.1186/s12877-016-0339-6

**Published:** 2016-09-27

**Authors:** Misa Nakamura, Yosuke Kobashi, Hiroshi Hashizume, Hiroyuki Oka, Ryohei Kono, Sachiko Nomura, Akihiro Maeno, Munehito Yoshida, Hirotoshi Utsunomiya

**Affiliations:** 1 Department of Rehabilitation, Osaka Kawasaki Rehabilitation University, 158 Mizuma, Kaizuka, Osaka 597-0104 Japan; 2 Department of Rehabilitation Medicine, Wakayama Medical University, 811-1 Kimiidera, Wakayama, Wakayama 641-8510 Japan; 3 Department of Orthopedic Surgery, Wakayama Medical University, 811-1 Kimiidera, Wakayama, 641-8510 Japan; 4 Department of Medical Research and Management for Musculoskeletal Pain, 22nd Century Medical & Research Center, Faculty of Medicine, University of Tokyo, Hongo 7-3-1, Bunkyo-ku, Tokyo, 113-8655 Japan; 5 Department of Strategic Surveillance for Functional Food and Comprehensive Traditional Medicine, Wakayama Medical University, Kimiidera 811-1, Wakayama, Wakayama 641-0012 Japan; 6 Laboratory of Chemistry, Kansai Medical University, 2-5-1 Shinnmachi, Hirakata, 573-1010 Japan

**Keywords:** Body composition, Locomotive syndrome, Bone mass index, Cardiometabolic disorders

## Abstract

**Background:**

A concept referred to as locomotive syndrome (LS) was proposed by the Japanese Orthopaedic Association in order to help identify middle-aged and older adults who may be at high risk of requiring healthcare services because of problems associated with locomotion. Cardiometabolic disorders, including obesity, hypertension, diabetes, and dyslipidemia, have a high prevalence worldwide. The purpose of this study was to determine the associations between LS and both body composition and cardiometabolic disorders.

**Methods:**

The study participants were 165 healthy adult Japanese women volunteers living in rural areas. LS was defined as a score ≥16 on the 25-question Geriatric Locomotive Function Scale (GLFS-25). Height, body weight, body fat percentage, body mass index (BMI), and bone status were measured. Bone status was evaluated by quantitative ultrasound (i.e., the speed of sound [SOS] of the calcaneus) and was expressed as the percent of Young Adult Mean of the SOS (%YAM). Comorbid conditions of hypertension, hyperlipidemia, and diabetes were assessed using self-report questionnaires.

**Results:**

Twenty-nine participants (17.6 %) were classed as having LS. The LS group was older, shorter, and had a higher body fat percentage, a higher BMI, and lower bone status than the non-LS group. Multiple logistic regression analysis showed that participants with a BMI ≥23.5 kg/m^2^ had a significantly higher risk for LS than those with a BMI <23.5 kg/m^2^ (odds ratio [OR] = 3.78, *p* < 0.01). Furthermore, GLFS-25 scores were higher in participants with than those without hypertension, diabetes, or obesity, and significantly increased with the number of present disorders.

**Conclusions:**

These findings suggest that BMI may be a useful screening tool for LS. Furthermore, because hypertension and diabetes were associated with LS, the prevention of these disorders accompanied by weight management may help protect against LS.

**Electronic supplementary material:**

The online version of this article (doi:10.1186/s12877-016-0339-6) contains supplementary material, which is available to authorized users.

## Background

Japan is rapidly transforming into a super-aged society. The Japanese Statistics Bureau reported that as of 2015, individuals aged 65 years or older comprised 26.2 % of the Japanese population [1]. Parallel with this transformation is an increase in the incidence of health issues such as stroke, senility, dementia, falls, fractures, and joint disorders, and in turn, the number of individuals requiring nursing care [2].

Maintaining a healthy locomotive system, which includes the bone, cartilage, muscle, and nervous systems, is the foundation of increased disability-free life expectancy. It follows that, from a public health perspective, preventing the deterioration of motor function is an issue that requires urgent attention. Therefore, an epidemiological concept referred to as locomotive syndrome (LS) [2, 3] has been proposed by the Japanese Orthopaedic Association (JOA). LS primarily affects elderly individuals who currently require nursing care services owing to problems involving the locomotive system or those who have risk conditions that will likely necessitate such services in the future [4]. LS is caused by the reduced muscle strength and balance associated with aging and locomotive pathologies including osteoporosis, osteoarthritis (OA), and sarcopenia [2]. In females, LS may also be caused by the decreasing levels of physical activity and bone mineral density (BMD) that tend to occur after menopause. The incidence of LS increases with age, and is significantly higher in women (35.6 %) than in men (21.2 %) [5, 6]. As beneficial locomotive exercises for the prevention of LS, the JOA recommends performing “half-squats” and “unipedal standing balance exercises with open eyes” [3].

The 25-question Geriatric Locomotive Function Scale (GLFS-25), a quantitative and evidence-based screening tool, can be used to identify individuals with LS [7, 8]. A previous study reported finding a Spearman’s correlation coefficient of 0.85 (*p* < 0.001) for the association between GLFS-25 scores and the European Quality of Life 5 Dimensions Index (EQ-5D), indicating that the GLFS-25 had excellent concurrent validity [7].

Identifying factors associated with the development of LS is crucial for its prevention. Results from a number of recent studies suggest that GLFS-25 scores strongly correlate with several physical performance measures, including the unipedal standing balance and Timed Up And Go tests [5, 9–13]. However, only a few reports [5, 14] have focused on the association between the development of LS and body composition, even though numerous studies have reported that weight, body fat percentage, and BMD are associated with cardiovascular disease, various cancers, osteoporosis, hypertension, diabetes and hyperlipidemia [15–21].

An association was recently reported between abdominal obesity and LS in elderly Japanese women, suggesting that waist circumference may be useful measure to assess the risk for LS [22].

Metabolic syndrome comprises a combination of medical disorders, including increased fasting plasma glucose, abdominal obesity, high triglyceride levels, and high blood pressure, that increase the risk of developing metabolic conditions and cardiovascular disease [23]. In conjunction with metabolic syndrome, obesity, hypertension, diabetes, and dyslipidemia, which are known as the “deadly quartet,” have a high prevalence worldwide [24]. Many studies have reported that physical activity and body components are associated with metabolic syndrome [17–21]. The proportion of the Japanese population with LS (47 million) is estimated to be more than twice that with metabolic syndrome (20 million) [25, 26].

In the present study, we evaluated body composition using body mass index (BMI), body fat percentage, and bone status, and hypothesized that these variables would be predictive of GLFS-25 scores. Confirmation of this hypothesis would indicate that, in addition to sports performance training, control of body composition could be used to prevent LS. Therefore, the purpose of this study was to determine the association between body composition and LS, the threshold values of body composition measures for discriminating between individuals with and without LS, and the OR of LS according to body composition above or below these thresholds in elderly Japanese women living in rural areas. An additional objective was to determine the association between LS and cardiometabolic disorders.

## Methods

### Participants

This study was conducted in a rural area (Tanabe city, Wakayama Prefecture, Japan) between January 2013 and March 2015. The study inclusion criteria were as follows: 1) Japanese women, age > 60 years; 2) ability to walk independently; and 3) living at home and being capable of self-care. All participants initially underwent body composition measurements, in the order of height, weight, and body fat percentage, followed by an evaluation of LS status at a public hall where a “Lecture meeting and checkup for health” was held with support from the local government. Afterwards, 198 women were asked to complete a self-report questionnaire at home regarding comorbid conditions and then to return the questionnaire by mail. A stamped envelope was provided to encourage the return of the questionnaire. A total of 165 women underwent the measurements and returned the questionnaire (mean age ± standard deviation, 68.8 ± 6.1 years; range, 60–83 years).

### Measurement of variables

Body weight and body fat percentage were measured using the KaradaScan Body Composition Monitor with Scale (HBF-362; Omron Co., Kyoto, Japan) while participants were wearing normal indoor clothing. The procedure was performed with the participants standing barefoot on a metal surface in accordance with the manufacturer’s instructions. BMI was calculated as weight divided by the square of height, and obesity was defined as BMI ≥25 kg/m^2^ in accordance with the guidelines of the Japan Society for the Study of Obesity [27]. Bone status was assessed using speed of sound (SOS) measured using a quantitative ultrasound (QUS) device (Canon Life Care Solutions Inc., CM-200, Osaka, Japan) at the calcaneus of the dominant foot while the participants were barefoot and seated. QUS, which has a number of advantages, including portability, low cost, and a lack of exposure to radiation [28], enables the evaluation of bone quality, especially the microarchitecture at the calcaneus, and is useful for predicting risk for future fracture [29]. Bone status was shown as the percent of Young Adult Mean of the SOS (%YAM) [30]. It has been reported that calcaneal QUS parameters reflect the characteristics of the trochanteric area of the proximal hip, although these values are not specifically reflective of values of the femoral neck or shaft [31].

### Evaluation of LS

LS status (presence and degree) was evaluated based on GLFS-25 scores ﻿(Additional file 1). The GLFS-25 is a self-report questionnaire composed of the following 25 items focusing on the month before completing the measure: four questions on pain; 16 on activities of daily living; three on social function; and two on mental health status [7]. These 25 items are scored from 0 (no impairment) to 4 (severe impairment), with a total score range from 0 to 100. Higher scores indicate worse locomotive function. The cutoff score for LS, as determined by receiver operating characteristic (ROC) analysis, is 16 points [7].

### Comorbid conditions

Comorbid conditions of hypertension, hyperlipidemia, and diabetes were assessed using the following question on the self-report questionnaire: “Do you presently take medication for hypertension, diabetes, or hyperlipidemia?”

### Statistical analysis

Participants were classified as LS (≥16) or non-LS (<16) based on GLFS-25 scores, and then independent variables were compared between groups. For numerical variables, normality of distribution and homogeneity of variance were tested before across-group comparisons. When the assumptions of normal distribution and homogeneity of variance were met in both groups, we performed the student t-test, and when the assumption of normal distribution was met, but not the assumption of homogeneity of variance, we performed Welch’s t-test. When the data were non-normally distributed, the Wilcoxon signed-rank test was used.

ROC analysis was used to evaluate the threshold of each body composition measure (BMI, body fat percentage, and %YAM) in order to discriminate the LS from the non-LS group. An area under the ROC (AUC-ROC) curve of 1.00 was taken to indicate perfect discrimination, whereas an AUC-ROC of 0.50 was taken to indicate the complete absence of discrimination.

Multiple logistic regression analysis was performed to evaluate the age-adjusted significance of the prevalence of LS. The chi-square test was used for comparison of prevalence or number of cardiometabolic disorders between non-LS and LS. The Wilcoxon signed-rank test was used for comparison of GLFS-25 scores classified by with and without cardiometabolic disorders, as well as by the number of present disorders. Statistical analysis was conducted using JMP 11 (SAS Institute, Cary, NC). All statistical tests were 2-tailed, and a significance level of 0.05 was used.

## Results

Age, height, body weight, body fat percentage, BMI, bone status, GLFS-25 score, and the prevalence of components of cardiometabolic disorder are shown in Table 1. Twenty-nine participants (17.5 %) had a GLFS-25 score ≥16 and were thereby classified as LS (Table 2). The LS group was older and shorter than the non-LS group, and had a higher body fat percentage, a higher BMI, and lower bone status (Table 2).

**Table 1:** Characteristics of the study participants

Variables for components	Mean (SD^a^)
Age (years)	68.8 (6.1)
Height (cm)	150.4 (11.9)
Weight (kg)	52.9 (8.3)
Body fat percentage (%)	33.9 (4.5)
BMI (kg/m^2^)	23.1 (3.6)
%YAM^b^ (%)	69.8 (11.0)
GLFS-25 score (points)	10.0 (10.3)
Components of cardiometabolic disorders	Prevalence (%)
Obesity	45 (27.3)
Hypertension	59 (35.8)
Diabetes mellitus	12 (7.3)
Hyperlipidemia	23 (13.9)

^a^ *SD* standard deviation

^b^ *YAM* percent of Young Adult Mean of the speed of sound of the calcaneus

**Table 2:** Comparison of characteristics between non-locomotive and locomotive syndrome^a^

Variables	Non-LS^b^ (*n* = 136)	LS^c^ (*n* = 29)	*p* value
Age (years)	68.1(5.9)	72.1(6.0)	0.0014^e^
Height (cm)	151.9(5.0)	143.7(25.5)	0.0015^e^
Weight (kg)	52.3(8.3)	55.4(8.3)	0.0730^f^
Body fat percentage (%)	33.4(4.3)	36.3(4.6)	0.0020^e^
BMI (kg/m^2^)	22.7(3.1)	25.2(3.7)	0.0007^e^
%YAM^d^ (%)	70.6(11.4)	65.7(8.3)	0.0288^f^

^a^Locomotive Syndrome: GLFS-25 score ≥16 points

^b^ *Non-LS* non-locomotive syndrome

^c^ *LS* locomotive syndrome

^d^ *YAM* percent of Young Adult Mean of the speed of sound of the calcaneus

^e^Wilcoxon signed-rank test was applied for age, height, body fat percentage, and BMI

^f^Student’s t-test was applied for weight and YAM

ROC analysis was conducted for each body composition measure, and the threshold for discriminating the non-LS and LS groups was identified. This threshold was 37.3 % for body fat percentage, 23.5 kg/m^2^ for BMI, and 73 % for %YAM (Table 3). ORs for the prevalence of LS according to the threshold values are shown in Table 4. High BMI was a significant risk factor for LS, with an OR of 3.78 as determined by multiple logistic regression analysis.

**Table 3:** Threshold values of age and body composition for locomotive syndrome

	Threshold values	AUC^a^	Sensitivity (%)	Specificity (%)
Body fat percentage (%)	37.3	0.68	51.72	68.13
BMI (kg/m^2^)	23.5	0.70	72.41	67.29
%YAM^b^ (%)	73.0	0.61	86.21	79.23

^a^ *AUC* area under the curve

^b^ *YAM* percent of Young Adult Mean of the speed of sound of the calcaneus

**Table 4:** Evaluation of odds ratios for locomotive syndrome according to body composition

	Above or below the threshold value	Odds ratio (95 % CI^a^)	*P* value
Body fat percentage (%)	<37.3	1	0.3584
	≥37.3	1.62 (0.58–5.00)	
BMI (kg/m^2^)	<23.5	1	0.0087
	≥23.5	3.78 (1.39–11.07)	
%YAM^b^ (%)	<73	1.68 (0.65–4.73)	0.2900
	≥73	1	

^a^ *CI* confidence interval

^b^ *YAM* percent of Young Adult Mean of the speed of sound of the calcaneus

Data were adjusted by age

**Fig. 1:**
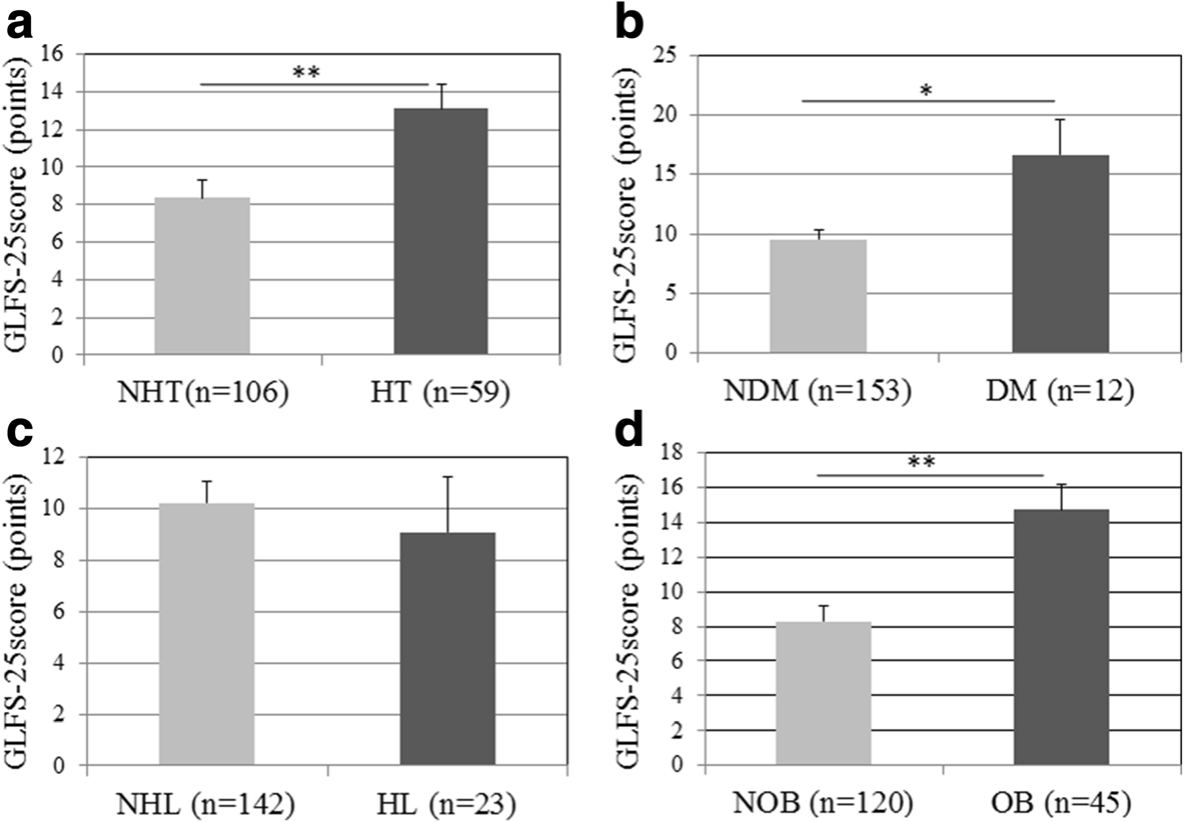
GLFS-25 scores in participants with and without hypertension (**a**), diabetes (**b**), hyperlipidemia (**c**), and obesity (**d**). HT, hypertension; NHT, non-hypertension; DM, diabetes mellitus; NDM, non-diabetes mellitus; HL, hyperlipidemia; NHL, non-hyperlipidemia; OB, obesity (BMI ≥25 kg/m^2^); NOB, non-obesity (BMI <25 kg/m^2^); *p < 0.05; **p < 0.01; error bars, standard deviation

**Fig. 2:**
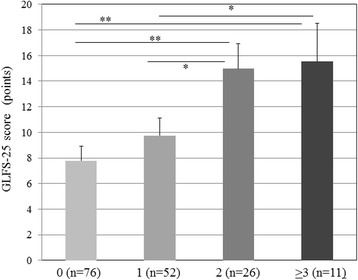
GLFS-25 scores in participants with 0, 1, 2, and ≥3 present cardiometabolic disorders. A significant main effect was observed for the number of disorders (*p* < 0.01; analysis of variance). *p < 0.05; **p < 0.01; error bars, standard deviation

Figure 1 shows GLFS-25 scores classified by the presence or absence of each metabolic syndrome component (hypertension, diabetes, hyperlipidemia, and obesity). GLFS-25 scores were higher in participants with than without hypertension or diabetes, and in obese than in non-obese participants. Figure 2 shows GLFS-25 scores classified by the number of present cardiometabolic disorders. The results showed that GLFS-25 scores significantly increased with the number of cardiometabolic disorders (*p* < 0.01). Table 5 shows a comparison of the prevalence or number of cardiometabolic disorders between non-LS and LS subjects. The prevalence of LS was higher in participants with than without hypertension (*p* < 0.05) and obesity (*p* < 0.01).

**Table 5:** Comparison of prevalence or number of present cardiometabolic disorders between non-locomotive and locomotive syndrome^a^

		Non-LS^b^	LS^c^	*P* value
NHT^d^ (153)		128 (83.7 %)	25 (16.3 %)	0.0482
HT^e^ (12)		8 (66.7 %)	4 (33.3 %)	
NDM^f^ (142)		116 (81.7 %)	26 (18.3 %)	0.1364
DM^g^ (23)		20 (87.0 %)	3 (13.0 %)	
NHL^h^ (106)		92 (86.8 %)	14 (13.2 %)	0.5382
HL^i^ (59)		44 (74.6 %)	15 (25.4 %)	
NOB^j^ (120)		106 (88.3 %)	14 (11.7 %)	0.0011
OB^k^ (45)		30 (66.7 %)	15 (33.3 %)	
Number of cardiometabolic disorders	0 (76)	67 (88.2 %)	9 (11.8 %)	0.0526
	1 (52)	44 (84.6 %)	8 (15.4 %)	
	2 (26)	18 (69.2 %)	8 (30.8 %)	
	≥3 (11)	7 (63.6 %)	4 (36.4 %)	

^a^Locomotive Syndrome: GLFS-25 score ≥16 points; ^b^ *Non-LS* non-locomotive syndrome, ^c^ *LS* locomotive syndrome, ^d^ *NHT* non-hypertension, ^e^ *HT* hypertension, ^f^ *NDM* non-diabetes, ^g^ *DM* diabetes, ^h^ *NHL* non-hyperlipidemia, ^i^ *HL* hyperlipidemia, ^j^ *NOB* non-obesity (BMI <25 kg/m^2^), ^k^ *OB* obesity (BMI ≥25 kg/m^2^)

## Discussion

### Association between body composition and LS

LS was proposed by the JOA in 2007 in order to identify individuals at high risk of requiring nursing care owing to problems associated with the locomotive system [2]. The GLFS-25 was subsequently developed to measure the presence and degree of LS in Japanese individuals [7]. However, since its implementation, the GLFS-25 cutoff value for identifying individuals with LS has been determined in accordance with health-related quality of life [7, 11]; therefore, information on the association between GLFS-25 scores and body composition is limited. Therefore, the primary purpose of this study was to determine the association between LS as defined by GLFS-25 scores and body composition measures in elderly Japanese women.

Our results showed that participants with LS were shorter, had a higher body fat percentage, a higher BMI, and lower bone status than participants without LS. Previous studies have reported similar results in middle-aged and elderly Japanese women [5, 14]. Muramoto et al. found that GLFS-25 scores had a significant positive correlation with body fat percentage and BMI, a negative correlation with body height and BMD, and no correlation with body weight according to correlation analysis [5].

Based on comparative analysis, participants with LS have been shown to have significantly greater BMI and body fat percentage and lower height than those without LS, whereas no significant difference has been observed in body weight or BMD [14].

In the present study, we found that participants with LS were shorter than those without LS. Shorter height has been reported to be significantly associated with fear of falling in elderly Japanese individuals [32]. Shorter height may be caused by an age-related change in the curvature of the spine or atrophy of trunk extension muscles, which can decrease postural control. A reduction in postural control can cause fear of falling or a decline in the amount of physical activity [33]. Therefore, we propose that the LS group included more participants that had lost height due to a change in the curvature of the spine or atrophy of trunk extension muscles than the non-LS group, and therefore had less postural control and engaged in fewer activities in daily life, which increased their risk for developing LS.

The present results showed that participants with LS had a higher body fat percentage than those without LS. Increased body fat causes more mechanical stress in weight-bearing joints and promotes the degeneration of joint tissue through the production and release of adipokines [34]. Adipokines are derived from adipocytes and may upregulate receptor activators of nuclear kappa B ligand, leading to increased bone resorption and reduced BMD [35]. Participants with a higher body fat percentage may have secreted more adipocytes, and this may have had a negative influence on the movement of the joints, thereby increasing the risk of LS.

The present study showed that a BMI ≥23.5 k/m^2^ was significantly associated with LS, with an OR of 3.78 as identified by multiple logistic regression analysis. The Japan Society for the Study of Obesity defines the cutoff for obesity as a BMI 25 kg/m^2^ [8]. In the present study, the mean BMI of the participants with LS was ≥25 kg/m^2^. Furthermore, GLFS-25 scores were higher in obese than in non-obese participants (Fig. 2d). LS is closely associated with age-related skeletal disorders such as osteoporosis, OA, lumbar spinal stenosis (LSS), degenerative spinal disease and sarcopenia [4]. Furthermore, obesity is a risk factor for these disorders because mechanical overload on weight-bearing joints can activate chondrocytes, accelerate the degeneration of cartilage, and increase static compressive loading and pressures associated with postures that damage disc integrity [36–38]. Moreover, it has been proposed that metabolic factors, including inflamed adipose tissue, dyslipidemia, oxidative stress, endothelial dysfunction and leptin dysregulation, as well as the clustering of these factors in metabolic syndrome, may play a crucial role in obesity-induced OA [39–41]. These findings support the present results regarding the association between obesity and LS.

### Association between LS and cardiometabolic disorders

Obesity, hypertension, diabetes, and dyslipidemia are known as the “deadly quartet” [24]. Numerous studies have reported that BMD is associated with these disorders [17–21]. The second purpose of the present study was to determine the association between LS and cardiometabolic disorders. Our results showed that LS is associated with hypertension, diabetes, and overweight, as well as with higher BMI. Furthermore, GLFS-25 scores significantly increased with the number of present cardiometabolic disorders.

There are some reports on the association between cardiometabolic disorders and OA. Some evidence suggests that metabolic factors such as type 2 diabetes mellitus and elevated glucose concentration are associated with the development and progression of OA [40, 42]. In particular, the advanced glycation end products in cartilage collagen seem to be associated with both the senescent cartilage matrix and reduced chondrocyte function [43]. The presence of advanced glycation end products associated with the expression of advanced glycation end-product receptors in the cartilage collagen results in the increased production of matrix metalloproteinase and the modulation of the chondrocyte phenotype to hypertrophy and OA [44, 45].

OA and hypertension have been shown to frequently coexist [46]. The proposed mechanism of the development of OA with hypertension is as follows: narrow and/or constricted vessels restrict blood flow to subchondral bone, impairing circulation and nutritional supply to overlying articular cartilage, which ultimately contributes to the deterioration of cartilage in OA [47].

Mutual relations exist between the occurrence and presence of musculoskeletal diseases, particularly knee OA and cardiometabolic disorders [48]. Yoshimura et al. suggested that metabolic risk factors such as overweight, hypertension, hyperlipidemia, and impaired glucose tolerance increase the risk of occurrence and progression of knee OA [49, 50]. Recent reports have indicated that waist circumference, back muscle strength, and spinal inclination angle are important risk factors for LS [22]. In the present study, we demonstrated that LS is associated with hypertension and obesity, as well as a higher BMI. Furthermore, GLFS-25 scores significantly increased with the number of present cardiometabolic disorders. These findings suggest a close relationship between the locomotive system and cardiometabolic organs.

The proportion of adults with BMI >25 kg/m^2^ has significantly increased worldwide [51]. The present findings contribute to the identification of factors that may prevent locomotive disorder and metabolic syndrome, particular in Western societies, in which many patients have metabolic syndrome. Although the concept of LS is currently used only in Japan, we believe it will become more common worldwide as the population continues to age.

The results of the present study suggest that BMI might be a useful measure for the simple detection of LS. Furthermore, hypertension and diabetes were found to be associated with LS. Weight management and prevention of these disorders may help protect against LS in elderly women. Elderly men should be included in future studies.

### Limitations and future research

This study did have several limitations. First, the sample size of 165 was small; this number only represents about 1.2 % of all women aged between 60 and 83 years in Tanabe city. Furthermore, no significant relationship was found between LS and dyslipidemia; this may have been due in part to a lack of statistical power. Second, because the participants in this study were all Japanese women, care should be taken in generalizing the results to men or other ethnic groups. Third, data from a cross-sectional study are not sufficient to determine whether a causal relationship exists between BMI, LS, and cardiometabolic disorders. LS may cause obesity or hypertension and diabetes because it limits physical activity. Conversely, these cardiometabolic disorders may lead to the development of LS. It is therefore crucial to perform longitudinal studies to clarify the causal relationships among these factors. Fourth, comorbid conditions were only assessed using self-report questionnaires; therefore, blood pressure, blood glucose concentration, and blood lipid concentration measurements were not controlled. Thus, untreated participants with comorbid conditions may have been excluded from analysis; however, this possibility is low because participants attending the “Lecture meeting” would have been expected to have relatively high health awareness. Fifth, further research in larger-sized studies should measure lean mass because it is an important component of BMI. It is possible that BMI underestimates body fat percentage in clinical populations [52].

## Conclusion

BMI, body fat percentage, and bone status were significantly associated with LS. In particular, a BMI ≥23.5 k/m^2^ was significantly associated with LS. Moreover, GLFS-25 scores were higher in participants with a BMI ≥25 kg/m^2^, hypertension, and diabetes than in the respective comparison groups. These results suggest that BMI is an important measure for the detection of LS. Furthermore, weight management and the prevention of metabolic syndrome may reduce the risk for LS.

## Additional file

Additional file 1: The 25-question Geriatric Locomotive Function Scale [7]. (DOCX 154 kb)

## Abbreviations

%YAM: Percent of Young Adult Mean of the SOS
AUC: area under the curve
BMD: Bone mineral density
BMI: Body mass index
GLFS-25: 25-question Geriatric Locomotive Function Scale
JOA: Japanese Orthopaedic Association
LS: Locomotive syndrome
OA: Osteoarthritis
OR: odds ratio
QUS: qualitative ultrasound
ROC: Receiver operating characteristic
SOS: speed of sound
