# Supplementary material for: Locomotive syndrome is associated with body composition and cardiometabolic disorders in elderly Japanese women
Source: BMC Geriatr. 2016 Sep 27;16:166. doi: 10.1186/s12877-016-0339-6 (PMC5039907; doi:10.1186/s12877-016-0339-6)
Supplement: Additional file 1: — The 25-question Geriatric Locomotive Function Scale [7]. (DOCX 154 kb) [file 12877_2016_339_MOESM1_ESM.docx]

The 25-question Geriatric Locomotive Function Scale [7]

The following questions are asking about your health status and usual daily life, relating to the involvement of your back and limbs. Please answer on your status ‘over the last one month’.

Following are questions about your body pain for the last one month:

1. Did you have any pain (including numbness) in your neck or upper limbs (shoulder, arm, or hand)?

No pain Mild pain Moderate pain Considerable pain

Severe pain

2. Did you have any pain in your back lower back or buttocks?

No pain Mild pain Moderate pain Considerable pain

Severe pain

3. Did you have any pain (including numbness) in your lower limbs (hip, thigh, knee, calf, shin, ankle, or foot)?

No pain Mild pain Moderate pain Considerable pain

Severe pain

4. To what extent has it been painful to move your body in daily life?

No pain Mild pain Moderate pain Considerable pain

Severe pain

Following are questions about your usual daily life for the last one month:

5. To what extent has it been difficult to get up from a bed or lie down?

Not difficult Mildly difficult Moderately difficult

Considerably difficult Extremely difficult

6. To what extent has it been difficult to stand up from a chair?

Not difficult Mildly difficult Moderately difficult

Considerably difficult Extremely difficult

7. To what extent has it been difficult to walk inside the house?

Not difficult Mildly difficult Moderately difficult

Considerably difficult Extremely difficult

8. To what extent has it been difficult to put on and take off shirts?

Not difficult Mildly difficult Moderately difficult

Considerably difficult Extremely difficult

9. To what extent has it been difficult to put on and take off trousers and pants?

Not difficult Mildly difficult Moderately difficult

Considerably difficult Extremely difficult

10. To what extent has it been difficult to use the toilet?

Not difficult Mildly difficult Moderately difficult

Considerably difficult Extremely difficult

11. To what extent has it been difficult to wash your body in the bath?

Not difficult Mildly difficult Moderately difficult

Considerably difficult Extremely difficult

12. To what extent has it been difficult to go up and down stairs?

Not difficult Mildly difficult Moderately difficult

Considerably difficult Extremely difficult

13. To what extent has it been difficult to walk briskly?

Not difficult Mildly difficult Moderately difficult

Considerably difficult Extremely difficult

14. To what extent has it been difficult to keep yourself neat?

Not difficult Mildly difficult Moderately difficult

Considerably difficult Extremely difficult

15. How far can you keep walking without rest? (please select the closet answer)

More than 2-3 km approximately 1 km approximately 300m

approximately 100m approximately 10m

16. To what extent has it been difficult to go out to visit neighbors?

Not difficult Mildly difficult Moderately difficult

Considerably difficult Extremely difficult

17. To what extent has it been difficult to carry objects weighing approximately 2 kilograms (2 standard milk bottles or 2 PET bottles each containing 1 liter)?

Not difficult Mildly difficult Moderately difficult

Considerably difficult Extremely difficult

18. To what extent has it been difficult to go out using public transportation?

Not difficult Mildly difficult Moderately difficult

Considerably difficult Extremely difficult

19. To what extent have simple tasks and housework (preparing meals, cleaning up, etc.) been difficult?

Not difficult Mildly difficult Moderately difficult

Considerably difficult Extremely difficult

20. To what extent have load-bearing tasks and housework (cleaning the yard, carrying heavy bedding, etc.) been difficult?

Not difficult Mildly difficult Moderately difficult

Considerably difficult Extremely difficult

21. To what extent has it been difficult to perform sports activity (jogging, swimming, gate ball, dancing, etc)?

Not difficult Mildly difficult Moderately difficult

Considerably difficult Extremely difficult

22. Have you been restricted from meeting your friends?

Not restricted Slightly restricted Restricted about half the time

Considerably restricted Gave up all activities

23. Have you been restricted from joining social activities (meeting friends, playing sport, engaging in activities and hobbies, etc.)?

Not restricted Slightly restricted Restricted about half the time

Considerably restricted Gave up all activities

24. Have you ever felt anxious about falls in your house?

Have not felt anxious Have occasionally felt anxious Have sometimes

felt anxious Have often felt anxious Have constantly felt anxious

25. Have you ever felt anxious about being unable to walk in the future?

Have not felt anxious Have occasionally felt anxious Have sometimes

felt anxious Have often felt anxious Have constantly felt anxious
